# Méningo-encéphalite lymphocytaire hypoglycorachique : au-delà de la tuberculose, penser à la neurobrucellose

**DOI:** 10.48327/mtsi.v6i3.2026.752

**Published:** 2026-09-25

**Authors:** Wiam AIT DRISS, HAJAR LAMHAMDI, Rania EL FARGANI, Malika I IDALENE, Noura TASSI

**Affiliations:** Service des maladies infectieuses – Centre hospitalier universitaire (CHU) Mohammed VI, Laboratoire de recherche de lutte contre les maladies infectieuses, Faculté de médecine et de pharmacie, Université Cadi, Ayyad, Marrakech, Maroc

**Keywords:** Neurobrucellose, *Brucella*, Méningo-Encéphalite, Méningite lymphocytaire, Sérodiagnostic, Maroc, Afrique du Nord, Neurobrucellosis, *Brucella*, Meningoencephalitis, Lymphocytic Meningitis, Serodiagnosis, Morocco, North Africa

## Abstract

**Introduction:**

La brucellose est une infection zoonotique commune dans de nombreux pays du monde causée par des bactéries du genre *Brucella*. La neurobrucellose peut se développer à n’importe quel stade de la maladie et peut avoir des manifestations très variables. L’objectif de ce travail est de souligner l’importance d’évoquer ce diagnostic devant tout tableau de méningo-encéphalite chronique dans un contexte d’exposition au risque brucellique.

**Patient et méthode:**

Nous rapportons le cas d’un patient de 65 ans, agriculteur, qui présentait un tableau de méningo-encéphalite d’évolution chronique depuis deux mois.

**Résultats:**

Les résultats de la ponction lombaire, de l’imagerie par résonnance magnétique et surtout la sérologie ont permis de conclure à une neurobrucellose. L’évolution sous bi-antibiothérapie a été favorable, avec régression des signes neurologiques et reprise de l’autonomie.

**Conclusion:**

La brucellose, bien que rare, doit rester dans les diagnostiques différentiels devant une méningite lymphocytaire hypoglycorachique, surtout en zone endémique.

## Abstract (en)

**Introduction:**

Brucellosis, a common zoonotic infection caused by bacteria of the genus *Brucella*, is prevalent in many countries around the world. Neurobrucellosis can develop at any stage of the disease and present with a variety of symptoms. This study aims to emphasize the importance of considering this diagnosis in cases of chronic meningoencephalitis when brucellosis exposure is a possibility.

**Patients and methods:**

We present the case of a 65-year-old farmer with a two-month history of chronic meningoencephalitis. **Results**. The results of the lumbar puncture, magnetic resonance imaging, and most importantly, serology, led to a diagnosis of neurobrucellosis. The patient responded well to dual antibiotic therapy, with regression of neurological signs and a return to independence.

**Conclusion:**

Although rare, brucellosis should remain in the differential diagnosis for hypoglycorrhachia lymphocytic meningitis, particularly in endemic regions.

**Figure:**
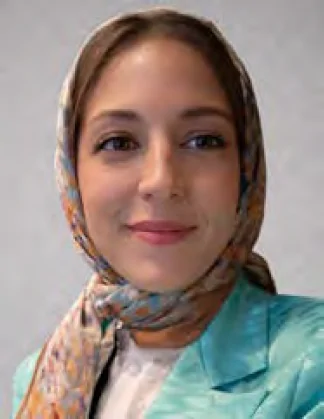

## Introduction

La brucellose est une zoonose ubiquitaire touchant en particulier les pays méditerranéens et le Moyen-Orient. Son incidence est en nette régression dans les pays développés alors qu’elle pose toujours un problème de santé publique dans les pays en voie de développement [12]. Elle est transmissible à l’homme à partir d’animaux réservoirs infectés. Les atteintes neurologiques peuvent être responsables de séquelles en cas de retard diagnostique et thérapeutique [1]. La neurobrucellose peut se développer à n’importe quel stade de la maladie avec des manifestations très variables, notamment une encéphalite, une méningo-encéphalite, une radiculite, une myélite, des neuropathies périphériques et crâniennes, une hémorragie sous-arachnoïdienne et des manifestations psychiatriques [12]. Dans la littérature, les critères diagnostiques de la neurobrucellose ne sont pas clairement standardisés. Leur manque de spécificité et la variabilité des présentations cliniques rendent le diagnostic parfois tardif ou erroné. Nous rapportons un cas de neurobru-cellose responsable d’une méningo-encéphalite traitée initialement comme tuberculose sans amélioration, dont le diagnostic a été posé devant un faisceau d’arguments épidémiologiques, cliniques, biologiques et radiologiques.

L’objectif de ce travail est de souligner les difficultés diagnostiques et la nécessité d’évoquer la neurobrucellose devant toute méningo-encépha-lite d’évolution prolongée en contexte épidémio-logique évocateur.

## Observation

Il s’agit d’un patient âgé de 65 ans, agriculteur de profession, résidant en milieu rural, avec notion de consommation régulière de produits laitiers non pasteurisés. Il a été hospitalisé dans le service des maladies infectieuses du CHU Mohammed VI au mois de mars 2025.

Depuis deux mois, il se plaignait de céphalées en casque associées à des vomissements en jet évoluant dans un contexte de fièvre et d’altération de l’état général associée à une asthénie et un amaigrissement non chiffré. L’évolution après deux mois a été marquée par l’installation de troubles de comportement à type d’agitation ayant motivé son hospitalisation.

À l’examen clinique, le patient était agité avec un score de Glasgow de 12/15. La fréquence cardiaque et respiratoire ainsi que la pression artérielle étaient normales, la température était à 39 °C. L’examen neurologique montrait une raideur de la nuque et un signe de Brudzinski. La station debout et la marche étaient difficiles à évaluer vu son état d’agitation. Le patient présentait aussi des myoclonies de l’hémicorps gauche en continu. L’examen pleuropulmonaire a objectivé des râles crépitants et des rhonchi basithoraciques à l’auscultation. Le reste de l’examen clinique était sans particularité.

Le scanner cérébral initial était interprété comme normal. La ponction lombaire révélait une pléiocytose à 389 éléments par mm3, à prédominance lymphocytaire (98 %); la glycorachie était à 0,26 g/l et la protéinorachie à 1,32 g/l. L’examen direct était négatif et la culture stérile. La PCR multiplex de type FilmArray réalisée sur le liquide céphalorachidien, permettant la détection rapide et simultanée de plusieurs agents responsables de méningites et d’encéphalites, incluant des bactéries, des virus et certains champignons, était négative.

Devant ce tableau clinique et biologique, le traitement anti-bacillaire associé à une corticothérapie a été initié le 9 mars. Par ailleurs, compte tenu de la notion de consommation de produits laitiers non pasteurisés, un traitement pour listériose à base de triméthoprime-sulfaméthoxazole (compte tenu de la non-disponibilité de l’amoxicilline) a été instauré en parallèle. Après obtention des résultats négatifs de la PCR multiplex FilmArray, le traitement anti-*Listeria* a été interrompu. En revanche, le traitement antibacillaire a été poursuivi, et les investigations étiologiques maintenues afin d’affiner le diagnostic.

Les examens ont été réalisés dans le liquide céphalorachidien (LCR), notamment le GeneXpert MTB/RIF, qui est une PCR en temps réel auto-matisée permettant la détection de l’ADN de *Mycobacterium tuberculosis* et la recherche de mutations associées à la résistance à la rifampicine. La culture du bacille de Koch (BK), le dosage de l’adénosine désaminase (ADA) et le QuantiFERON-TB Gold Plus (dosage sanguin permettant le diagnostic d’une infection tuber-culeuse latente), se sont révélés négatifs. Ces résultats, bien qu’à interpréter avec prudence, ne fournissaient pas d’arguments probants en faveur d’une méningo-encéphalite tuberculeuse. En revanche, la sérologie de la brucellose dans le sang, comprenant le test Rose Bengale par agglutination, est revenue positive. Associée à la forte positivité de la sérologie de Wright, cela constituait un argument décisif en faveur du diagnostic de neurobrucellose. Malheureusement, la sérologie de la brucellose dans le LCR n’était pas disponible.

La sérologie de la syphilis est revenue négative, ce qui a exclu la neurosyphilis. La sérologie VIH était également négative.

L’IRM encéphalique a montré des anomalies de signal cortico souscorticales sustentorielles bilatérales, en rapport avec une encéphalite infectieuse. Un électroencéphalogramme réalisé lors des myoclonies montrait un tracé symétrique avec des pointesondes concomitantes aux secousses musculaires en faveur d’une encéphalopathie infectieuse, un aspect généralement retrouvé chez tout patient atteint de brucellose avec des troubles de la conscience ou des crises comitiales. La numération formule sanguine (NFS) montrait une leucocytose à 21,57 G/l, à prédominance neutrophilique (17,25 G/l), tandis que la CRP était légèrement élevée à 30 mg/l. Le taux de plaquettes et le bilan hépatique étaient normaux, ce qui correspond aux constatations classiques observées au cours de la brucellose.

Les antibacillaires et la corticothérapie ont été arrêtés après trois semaines, mais la rifampicine à la posologie de 600 mg deux fois par jour a été maintenue.

L’évolution du patient a été favorable, avec reprise de la conscience, disparition progressive des myoclonies et récupération de l’autonomie. La régression des troubles cognitifs et des myoclonies a été constatée dès la première semaine de traitement. À la consultation de contrôle après un mois, une amélioration clinique supplémentaire a été observée avec une ponction lombaire (PL) de contrôle normale à la fin de traitement. La durée préconisée du traitement était de trois mois.

Plusieurs éléments nous ont orientés vers une neurobrucellose : le contexte de vie rurale du patient avec consommation de produits laitiers non pasteurisés, l’évolution clinique chronique, l’absence d’amélioration sous traitement initial par anti-bacillaires et corticothérapie et l’absence d’argument probant pour une autre pathologie. Le maintien de la rifampicine est justifié car il constitue le traitement de première intention dans la brucellose et fait partie du programme national de prise en charge. La rifampicine a été associée à la doxycycline à raison de 100 mg deux fois par jour 200 mg/j.

**Figure 1:**
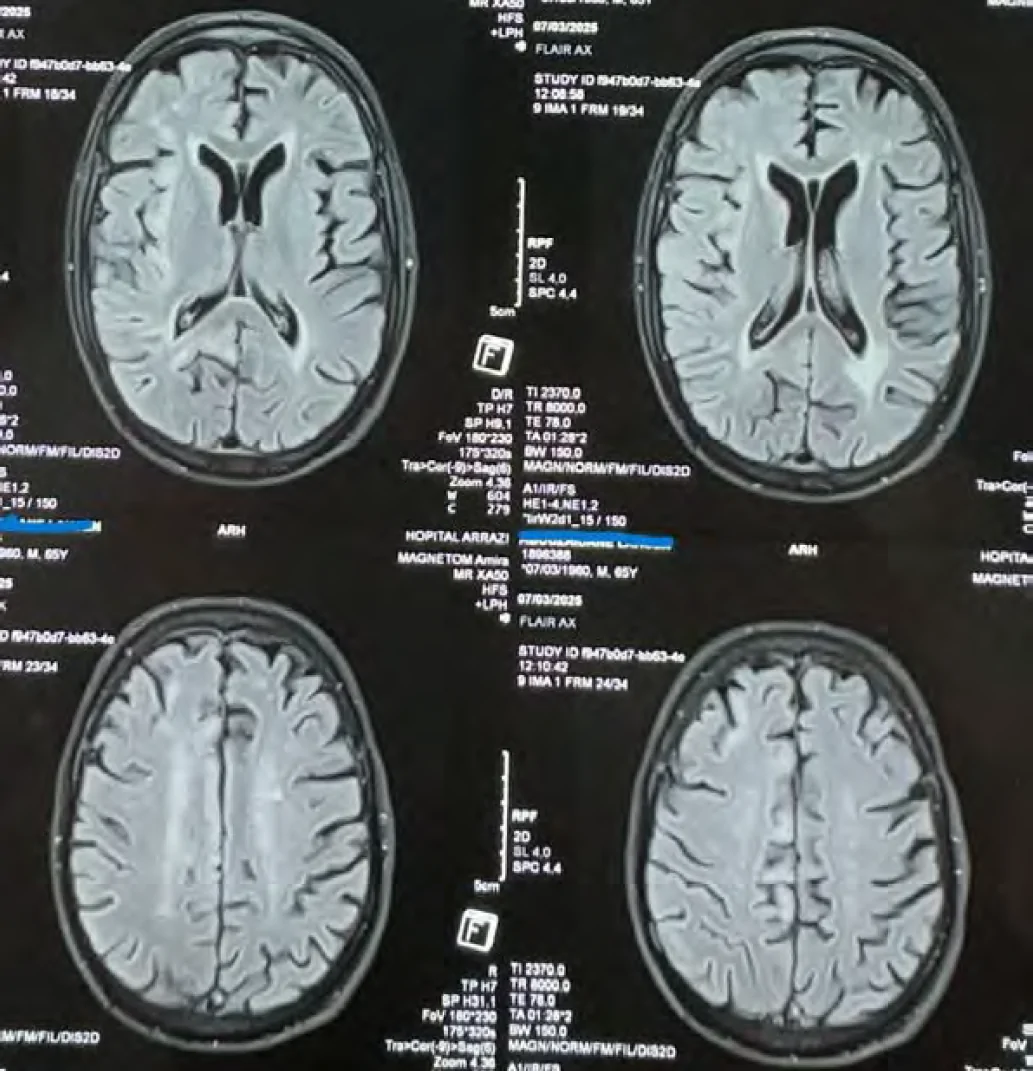
Imagerie par résonance magnétique cérébrale : anomalies de signal cortico sous corticales sus tentorielles bilatérales en rapport avec une encéphalite infectieuse

## Discussion

La brucellose est la zoonose ubiquitaire la plus répandue dans le monde avec plus d’un demi-million de nouveaux cas estimés chaque année [8]. Elle demeure endémique dans certains pays y compris le bassin méditerranéen et est encore considérée comme un problème de santé publique dans les pays en voie de développement [3]. Elle est souvent sousdiagnostiquée à cause de son polymorphisme clinique [7]. L’Afrique du Nord a toujours été considérée comme endémique pour la brucellose [9]. Au Maroc, elle est classée maladie à déclaration obligatoire depuis 1967. Sa séroprévalence est estimée à 1,5 % en milieu rural, selon la surveillance épidémiologique de routine et une enquête du ministère de la Santé menée en 1999 [10]. La brucellose est causée principalement par l’une des trois espèces du genre *Brucella* : *B. melitensis, B. abortus et B. suis*. Les principaux réservoirs d’agents pathogènes sont les bovins, les ovins et les caprins. La transmission à l’homme se fait principalement par contact direct avec le bétail, en général par voie cutanéo-muqueuse ou, indirectement, par voie digestive. La contamination est liée aux habitudes alimentaires (lait cru, fromage frais, crème non pasteurisée). La contamination interhumaine est exceptionnelle [12].

Les manifestations cliniques de la maladie sont peu spécifiques et son principal diagnostic différentiel dans notre contexte est la tuberculose. Elles peuvent survenir sur un mode aigu, ou être plus insidieuses. La brucellose se manifeste le plus souvent sous la forme d’un tableau pseudo grippal avec fièvre, sueurs, anorexie, asthénie, céphalées, myalgies et arthralgies. Son évolution spontanée se caractérise surtout par la possibilité de survenue de localisations secondaires qui font la gravité de la maladie. Cellesci peuvent être neuroméningées, cardiaques, ostéoarticulaires, hépatospléniques ou génitales [12]. Les formes chroniques se définissent par une évolution pro-longée audelà d’un an, avec ou sans découverte d’un foyer infectieux focalisé [12]. La neurobru-cellose représente une complication rare mais non exceptionnelle de la brucellose dans les régions d’endémie, avec une fréquence estimée entre 1,7 % et 10 % des cas selon les séries [1].

Sur le plan évolutif, l’atteinte neurologique peut survenir à la phase aiguë ou chronique de la maladie. Cette atteinte peut avoir des manifesta-tions variables centrales et/ou périphériques. La méningo-encéphalite est la présentation la plus fréquente de la neurobrucellose [6,11]. Ce qui est le cas chez notre patient.

Le diagnostic de neurobrucellose est souvent retardé de 2 à 12 mois après les premiers symp-tômes. Il repose sur un faisceau d’arguments épidémiologiques (exposition professionnelle), cliniques (signes neurologiques), radiologiques (anomalies du système nerveux central en IRM), biologiques (anomalies du LCR, mise en évidence du germe, sérologie brucellienne) et une réponse favorable au traitement [1,4,7].

L’isolement des *Brucella* en culture est la tech-nique de référence pour établir un diagnostic certain de brucellose. *Brucella* est souvent isolée du sang (hémoculture) et plus rarement du LCR (dans 30 à 50 % des cas) [13]. Il convient donc de sensibiliser les microbiologistes à cette éventualité et de recommander le prolongement de l’incu-bation des cultures lorsque le contexte clinique est évocateur, même en l’absence d’arguments microbiologiques initiaux. Une telle vigilance peut faire la différence entre un diagnostic établi et un diagnostic manqué.

Les méthodes sérologiques sont nombreuses. La technique de séroagglutination de Wright est la première technique sérologique décrite et demeure la référence préconisée par l’Organisation mondiale de la Santé du fait de sa standardisation. Les autres techniques sérologiques développées incluent la technique d’agglutination sur lame ou l’épreuve à l’antigène tamponné, la réaction de fixation du complément, la technique d’immunofluorescence indirecte, et les tests ELISA. Quel que soit le test sérologique, des réactions croisées sont observées avec des infections par *Yersinia enterolitica* O9, *Vibrio cholerae* et *Francisella tularensis* [13]. Chez notre patient, la sérologie de la brucellose dans le sang est revenue positive, mais malheureusement la sérologie de la brucellose dans le LCR n’était pas disponible. La PCR dans le LCR constitue un outil diagnostique complémentaire de plus en plus utile au cours de la neurobrucellose, permettant une détection rapide et spécifique de *Brucella* spp., notamment dans les situations où la culture est négative ou la sérologie peu contributive.

Le traitement de la neurobrucellose est basé sur des antibiotiques ayant une bonne diffusion à travers la barrière hémato-encéphalique et en intracellulaire. La résistance aux antibiotiques de *Brucella* est rare. Les antibiotiques les plus actifs sont les aminosides, les tétracyclines, la rifampicine et les fluoroquinolones. L’association d’au moins deux antibiotiques est la règle pendant une durée minimum de trois mois. Les deux associations les plus utilisées sont : rifampicine/triméthoprime sulfaméthoxazole ou rifampicine/doxycycline [2]. Les rechutes sont rares, surviennent généralement dans la première année du traitement et sont dues à une mauvaise observance du traitement [5]. Si le LCR est encore anormal, les patients doivent être traités pendant six mois, voire deux ans.

## Conclusion

La brucellose, bien que rare, doit rester dans le spectre des méningites lymphocytaires hypoglycorachiques, à l’instar de la tuberculose, surtout en zone endémique ou chez les sujets à risque. Le traitement repose sur une antibiothérapie adaptée, généralement basée sur des associations d’anti-biotiques afin de réduire le risque de rechute. Par ailleurs, la prévention constitue un élément clé du contrôle de la maladie à l’échelle populationnelle. Elle repose principalement sur la lutte contre la brucellose animale, notamment par les mesures sanitaires dans le domaine agricole et vétérinaire, ainsi que sur la sensibilisation à la consommation de produits laitiers pasteurisés.

## Financement de l’étude

Cette étude n’a reçu aucun financement.

## Consentement éclairé

Nous avons obtenu le consentement oral du patient.

## Contribution des auteurs et autrices

Wiam Ait Driss : conception et rédaction Hajar lamhamdi : collecte des données Rania el Fargani : recherche bibliographique Malika Idalene : relecture et révision

Noura Tassi : relecture et approbation de la version finale

## Conflits d’intérêts

Aucun lien d’intérêt n’a été déclaré.
