# Supplementary material for: Hypoglycorrhachia lymphocytic meningoencephalitis: beyond tuberculosis, consider neurobrucellosis
Source: Med Trop Sante Int. 2026 Sep 25;6(3):mtsi.v6i3.2026.752. [Article in French] doi: 10.48327/mtsi.v6i3.2026.752 (PMC13697908; doi:10.48327/mtsi.v6i3.2026.752)
Supplement: Related texts

# Research article: Hypoglycorrhachia lymphocytic meningoencephalitis: beyond tuberculosis, consider neurobrucellosis

## Introduction

Brucellosis is a widespread zoonotic disease that primarily affects countries in the Mediterranean region and the Middle East. While its incidence is declining sharply in developed countries, it remains a public health problem in developing countries [12]. The disease can be transmitted to humans from infected reservoir animals. If diagnosis and treatment are delayed, neurological involvement can lead to long-term sequelae [1]. Neurobrucellosis can develop at any stage of the disease and has highly variable manifestations, including encephalitis, meningoencephalitis, radiculitis, myelitis, peripheral and cranial neuropathies, subarachnoid hemorrhage, and psychiatric symptoms [12]. The diagnostic criteria for neurobrucellosis are not clearly standardized in the literature. The lack of specificity of these criteria and the variability of clinical presentations can lead to delayed or incorrect diagnoses. We present a case of neurobrucellosis presenting as meningoencephalitis that was initially misdiagnosed as tuberculosis and did not improve with treatment. The diagnosis was made based on a combination of epidemiological, clinical, laboratory, and radiological findings.

This study aims to highlight the diagnostic challenges and emphasize the importance of considering neurobrucellosis in cases of prolonged meningoencephalitis in epidemiologically suggestive contexts.

## Case report

The patient is a 65-year-old farmer who lives in a rural area and regularly consumes unpasteurized dairy products. In March 2025, he was admitted to the infectious diseases department of Mohammed VI University Hospital.

For the previous two months, he had complained of a band-like headache accompanied by projectile vomiting. This occurred alongside a fever, a decline in his general condition, asthenia, and unquantified weight loss. After two months, his condition was marked by the onset of behavioral disturbances, specifically agitation, which led to his hospitalization.

Upon physical examination, the patient appeared agitated and had a Glasgow Coma Scale score of 12/15. His heart rate, respiratory rate, and blood pressure were normal, and his temperature was 39°C. The neurological examination revealed nuchal rigidity and Brudzinski’s sign. Standing and walking were difficult to assess due to his agitation. The patient also presented with continuous myoclonus of the left side of the body. The pleuropulmonary examination revealed low-chest crackles and rhonchi on auscultation. The rest of the clinical examination was unremarkable. The initial brain CT scan was interpreted as normal. A lumbar puncture revealed pleocytosis of 389 cells/mm^³^, predominantly lymphocytic (98%). The cerebrospinal fluid glucose level was 0.26 g/L, and the protein level was 1.32 g/L. Direct examination was negative, and the culture was sterile. The FilmArray multiplex PCR test, which enables the rapid and simultaneous detection of several agents responsible for meningitis and encephalitis (including bacteria, viruses, and certain fungi), was negative when performed on the cerebrospinal fluid.

Based on the clinical and laboratory findings, a combination of anti-TB and corticosteroid treatments was initiated on March 9. Additionally, due to the patient’s history of consuming unpasteurized dairy products, treatment for listeriosis with trimethoprim-sulfamethoxazole was initiated concurrently, as amoxicillin was unavailable. After receiving negative results from the FilmArray multiplex PCR test, treatment for *Listeria* was discontinued. However, anti-TB treatment continued, and etiological investigations were maintained to refine the diagnosis.

Tests were performed on the cerebrospinal fluid (CSF), including the GeneXpert MTB/RIF assay, an automated, real-time PCR test that detects *Mycobacterium tuberculosis* DNA and screens for mutations associated with rifampin resistance. Cultures for Koch’s bacillus (KB), adenosine deaminase (ADA) testing, and QuantiFERON-TB Gold Plus (a blood test for diagnosing latent tuberculosis infection) were negative. While these results should be interpreted with caution, they did not conclusively support a diagnosis of tuberculous meningoencephalitis. However, brucellosis serology in the blood, including Rose Bengal agglutination testing, was positive. Combined with the strongly positive Wright serology, this constituted decisive evidence in favor of a neuro-brucellosis diagnosis. Unfortunately, brucellosis serology in the CSF was unavailable.

Syphilis serology was negative, ruling out neurosyphilis. HIV serology was also negative.

An MRI of the brain revealed bilateral supratentorial cortical and subcortical signal abnormalities consistent with infectious encephalitis. An electroencephalogram performed during myoclonic episodes showed symmetrical spike-and-wave patterns concurrent with muscle jerks, which is a finding commonly observed in patients with brucellosis who present with impaired consciousness or seizures and is consistent with infectious encephalopathy.

A complete blood count (CBC) showed leukocytosis at 21.57 G/L with neutrophilic predominance at 17.25 G/L, and a slightly elevated C-reactive protein (CRP) level of 30 mg/L. The platelet count and liver function tests were normal, consistent with classic brucellosis findings.

After three weeks, anti-TB and corticosteroid treatments were discontinued, but rifampin at a dose of 600 mg twice daily was continued.

The patient had a favorable outcome, regaining consciousness, gradually resolving myoclonus, and recovering independence. Improvement in cognitive impairment and myoclonus was noted as early as the first week of treatment. At the one-month follow-up visit, further clinical improvement was observed, and the follow-up lumbar puncture (LP) was normal at the end of treatment. The recommended treatment duration was three months.

We suspected neurobrucellosis due to several factors: the patient’s rural lifestyle involving consumption of unpasteurized dairy products; the chronic clinical course; the lack of improvement with initial treatment using anti-TB and cortico-steroid treatments; and the absence of compelling evidence for another condition.

Continuing rifampin treatment is justified because it is the primary treatment for brucellosis and part of the national treatment program. Rifampin was combined with doxycycline at a dose of 100 mg twice daily (200 mg/day).

**Figure 1:** Brain magnetic resonance imaging: bilateral supratentorial cortical and subcortical signal abnormalities associated with infectious encephalitis

## Discussion

Brucellosis is the most widespread zoonosis in the world, with an estimated half a million new cases each year [8]. It remains endemic in certain countries, including those in the Mediterranean basin, and it is still considered a public health problem in developing countries [3]. The disease is often underdiagnosed due to its clinical polymorphism [7]. North Africa has always been considered an endemic region for brucellosis [9]. Morocco has classified it as a notifiable disease since 1967. According to routine epidemiological surveillance and a 1999 survey conducted by the Ministry of Health, its seroprevalence is estimated at 1.5% in rural areas [10]. Brucellosis is primarily caused by one of three species of the genus *Brucella*: *B. melitensis*, *B. abortus*, and *B. suis.* The main reservoirs of these pathogens are cattle, sheep, and goats. Transmission to humans primarily occurs through direct contact with livestock via the skin or mucous membranes or indirectly through the digestive tract. Infection is linked to dietary habits, such as consuming raw milk, fresh cheese, and unpasteurized cream.

Human-to-human transmission is rare [12]. The clinical manifestations of the disease are non-specific, and the main differential diagnosis in this context is tuberculosis. Symptoms may present acutely or develop more insidiously. Brucellosis most often presents as flu-like symptoms, including fever, night sweats, loss of appetite, fatigue, headaches, muscle pain, and joint pain. The disease’s natural history is primarily characterized by the potential for secondary involvement in various sites, which determines its severity. These sites may include the nervous system, the heart, bones and joints, the liver and spleen, and the genitals [12]. Chronic forms are defined by a course lasting more than one year, with or without identification of a localized focus of infection [12]. Neurobrucellosis is a rare but not exceptional complication of brucellosis in endemic regions, with an estimated frequency ranging from 1.7% to 10%, depending on the study [1]. In terms of disease progression, neurological in-volvement may occur during the acute or chronic phase. These manifestations may affect the central and/or peripheral nervous systems and can vary. The most common presentation of neurobrucellosis is meningoencephalitis [6,11]. This was the case in our patient.

Diagnosis of neurobrucellosis is often delayed by two to 12 months after symptom onset. It is based on a combination of epidemiological factors (e.g., occupational exposure), clinical signs (e.g., neurological symptoms), radiological findings (e.g., central nervous system abnormalities on MRI), and laboratory results (e.g., CSF abnormalities, pathogen detection, brucellosis serology), as well as a favorable response to treatment [1,4,7].

Isolating *Brucella* in culture is the gold standard for definitively diagnosing brucellosis. *Brucella* is often isolated from blood (blood culture) and, less commonly, from CSF (in 30% to 50% of cases) [13]. Therefore, microbiologists should be aware of this possibility. It is recommended that culture incubation periods be extended when the clinical presentation suggests brucellosis, even in the absence of initial microbiological evidence. Such vigilance can mean the difference between a confirmed diagnosis and a missed one.

There are numerous serological methods. The Wright seroagglutination test was the first serological technique described, and it remains the gold standard recommended by the World Health Organization due to its standardization. Other techniques include slide agglutination, test with buffered antigen, complement fixation, indirect immunofluorescence, and ELISA. Regardless of the serological test used, cross-reactions are observed with infections caused by *Yersinia enterocolitica* O:9, *Vibrio cholerae*, and *Francisella tularensis* [13]. Our patient tested positive for brucellosis in a blood serology test. Unfortunately, CSF serology for brucellosis was unavailable. PCR in CSF is an increasingly useful diagnostic tool for neurobrucellosis, as it allows for the rapid and specific detection of *Brucella* spp., particularly when cultures are negative or serology is not helpful.

The treatment for neurobrucellosis involves antibiotics that diffuse well across the blood-brain barrier and exhibit intracellular activity. Resistance to antibiotics in *Brucella* is rare. The most effective antibiotics are aminoglycosides, tetracyclines, rifampin, and fluoroquinolones. Combination therapy with at least two of these antibiotics is standard for a minimum of three months. The two most commonly used combinations are rifampin/ trimethoprim-sulfamethoxazole and rifampin/ doxycycline [2].

Relapses are rare and generally occur within the first year of treatment due to poor treatment adherence [5]. Patients with abnormal CSF should be treated for six months or up to two years.

## Conclusion

Although brucellosis is rare, it must be considered among the causes of hypoglycorrhachia lymphocytic meningitis, just as tuberculosis is, especially in endemic areas or among high-risk individuals. Treatment relies on appropriate antibiotic therapy, typically involving combinations of antibiotics, to reduce the risk of relapse. Furthermore, prevention is key to controlling the disease at the population level. This primarily involves combating animal brucellosis through health measures in the agricultural and veterinary sectors and raising awareness about consuming pasteurized dairy products.

## Funding

This study received no funding.

## Informed consent

We obtained verbal consent from the patient.

## Authors’ Contributions

Wiam Ait Driss: Design and drafting Hajar Lamhamdi: Data collection Rania El Fargani: Literature review

Malika Idalene: Proofreading and revision Noura Tassi: Proofreading and approval of the final version

## Conflicts of interest

The authors declare no conflicts of interest.
